# Exploring the effectiveness of virtual and in-person instruction in culinary medicine: a survey-based study

**DOI:** 10.1186/s12909-024-05265-w

**Published:** 2024-03-13

**Authors:** Orli Glickman, Joanne Kakaty-Monzo, Michael Roberts, Farzaneh Daghigh

**Affiliations:** The Philadelphia College of Osteopathic Medicine, Philadelphia, United States

**Keywords:** Culinary medicine, Nutrition, Lifestyle, Chronic disease, Medical education, Diet

## Abstract

**Background:**

Culinary medicine, which has recently increased in popularity in medical education, incorporates food and nutritional interventions with principles of disease prevention and treatment. The ultimate goal is to improve overall health outcomes. The growing prevalence of diet-related chronic diseases indicates the need for physicians to have a deeper understanding of the interplay between nutrition and disease. Incorporating culinary medicine into medical education can equip medical students with the necessary skills and knowledge to promote better patient outcomes. The purpose of this study was to evaluate students’ perceptions of their foundational knowledge of a culinary medicine course after completion of the course for first- and second-year medical students at the PCOM (Philadelphia College of Osteopathic Medicine). We will also examine the difference between methods of instruction in relation to constructs discussed of knowledge gained and enjoyment of the course.

**Methods:**

This retrospective cohort study was conducted using SurveyMonkey by Momentive. Data were collected from osteopathic medical students who enrolled in a culinary medicine course at the PCOM from 2018 to 2022 through the completion of a post-course survey. The methods of instruction included either a virtual or in-person classroom. The statistical analysis for this study was conducted using IBM SPSS Statistics version 28. To compare methods of instruction, the statistical analyses used included descriptive statistics, chi-square analysis, one-way ANOVA, and independent sample one-sided t tests.

**Results:**

A total of 360 out of 430 participants, spanning the years 2018 to 2022, completed the course requirements and participated in the online survey. There was a valid sample size of 249 for the in-person group and 111 for the virtual instruction group. The knowledge gained construct consisted of five survey questions, for a total possible score of 25, while the enjoyment construct consisted of two questions, for a total possible score of 10. A statistically significant difference in knowledge gained was identified by one-way ANOVA, *F* (4,355) = 3.853, *p* =.004. Additionally, there was a statistically significant difference in enjoyment of the course between class years, *F* (4,356) = 11.977, *p* <.001. Independent sample t-tests revealed a statistically significant difference in enjoyment between the two methods (*p* <.001) even after accounting for unequal variances, with Cohen’s d equal to 0.807, indicating a moderate effect size.

**Conclusions:**

The findings of this study suggest that overall, students were highly satisfied with the Culinary Medicine course over a five-year period. The study suggested that students who participated in in-person courses benefitted more than did the virtual students in terms of knowledge gained and enjoyment. The 360 students who completed the Culinary Medicine course were highly satisfied with the information and skills they acquired.

**Supplementary Information:**

The online version contains supplementary material available at 10.1186/s12909-024-05265-w.

## Background

Culinary medicine, the intersection of the art of cooking and the science of medicine, has increased in popularity with medical education in recent years [1]. It incorporates food and nutritional interventions with principles of disease prevention and treatment, as well as improving overall health outcomes. The growing prevalence of diet-related chronic diseases such as diabetes, obesity, and heart disease indicates the need for physicians to have a deeper understanding of the interplay between nutrition and disease [2]. Incorporating culinary medicine into medical education can equip medical students with the necessary skills and knowledge to promote better patient outcomes [3].

Educational courses on culinary medicine typically involve didactic lectures, hands-on cooking sessions, and clinical experience [4]. Didactic lectures provide medical students with foundational knowledge on the role of nutrition in health and disease, including the impact of diet on chronic diseases [1]. Hands-on cooking sessions allow students to apply their nutritional and culinary skills by preparing healthy meals, which they can use to relate to patients when discussing cooking. Clinical experience provides opportunities for students to use their knowledge in real-life patient encounters to guide patients in lifestyle and diet changes for better disease management.

By implementing culinary medicine for future physician training, students learn to provide a patient-centered approach to care through the importance of nutritional and culinary skills in preventing and treating disease. Goldring et al. reported that medical students who participated in culinary medicine education had greater appreciation for the importance of nutrition in patient care and were more likely to incorporate nutritional counseling into their practice [5]. Another study by Black et al. demonstrated that medical students who participated in a culinary medicine program had improved confidence in their ability to counsel patients on nutrition and cooking [6]. Improved patient outcomes include improved glycemic control in patients with diabetes, reduced blood pressure in patients with hypertension, and improved cholesterol levels in patients with hyperlipidemia [7]. Culinary medicine education can help physicians provide more personalized and effective care by tailoring food and nutritional interventions to individual patients’ needs and preferences.

There are challenges in incorporating culinary medicine into medical education. One challenge is the limited amount of time available in the medical curriculum, as medical schools often have a crowded curriculum with little room for additional topics. Despite this challenge, some medical schools have found innovative ways to overcome it, such as integrating culinary medicine into clinical skills training [8]. Another challenge is the lack of standardized culinary medicine curricula. While there are several culinary medicine programs available, there is no standardization of the curriculum or assessment. This makes it difficult to compare the effectiveness of different culinary medicine programs and ensure that medical students receive the necessary knowledge and skills to provide effective nutritional counseling and cooking advice to patients.

The culinary teaching class faces potential limitations that may impact students’ experiences, such as challenges in delivering hands-on experiences in a virtual setting, variations in kitchen resources among students, and potential distractions affecting engagement. The requirement for virtual students to purchase their own ingredients, unlike in-person students, may present a barrier. Instructors can address these limitations through innovative teaching strategies, clear instructions, and incorporation of interactive elements to enhance engagement [9].

In this study, we aimed to determine the ideal method of instruction for a culinary medicine course for first- and second-year osteopathic medical students at the Philadelphia College of Osteopathic Medicine (PCOM). We will also examine the difference between methods of instruction in relation to constructs discussing the knowledge gained and the enjoyment of the course. The methods of instruction included either a virtual or in-person classroom setting.

### Hypothesis 1

Student participants in an in-person classroom setting will report more knowledge gained at the conclusion of the course than student participants in a virtual classroom.

### Hypothesis 2

Student participants in an in-person culinary medicine classroom will report more enjoyment of the course than student participants in a virtual classroom.

### Hypothesis 3

Students taking the course in the years 2020 and 2021, when the course shifted to virtual instruction due to the COVID-19 pandemic, will report lower levels of knowledge gained and enjoyment than student responses from the years 2018, 2019, and 2022.

## Methods

### Study design and participants

This retrospective cohort study was conducted using SurveyMonkey by Momentive, a website for creating, collecting, and analyzing survey responses. The data were collected from osteopathic medical students who were enrolled in the culinary medicine course at the PCOM. This sample was considered convenient because students voluntarily enrolled in the study and were subsequently included in the data collection as part of the course. The survey questions were self-generated specifically for this study based on the course objectives. Following the creation of the items, the measure was reviewed by topic experts, and each item agreed upon to measure the constructs of interest (i.e., knowledge gained and enjoyment). Further discussion of the psychometric properties of the measure is provided later. The inclusion criteria included being enrolled in and successfully completing four modules of the culinary medicine curriculum, which was offered twice for each class of students, once in the spring of their first year and once in the fall of their second year. Students applied voluntarily for the course using an interest form on Google Forms, and spots were allocated on a first-come, first-serve basis with options for multiple days per week. The elective course was worth one credit to a doctor of osteopathic medicine (D.O.) degree. Only students who completed the survey at the end of the course were included in the study analysis.

Two methods of instruction were used for the course: virtual through Blackboard Collaborate, using students’ personal accounts associated with PCOM, and in-person on campus. Course surveys from the years 2018 through 2022 were included in the data analysis. Due to the COVID-19 pandemic, data from 2020 and partially 2021 were collected using a virtual method of instruction, which included cooking demonstrations, lectures, and discussions through the Blackboard Collaborate, with students required to have their cameras on during the entire session. The cooking portion of the course was conducted with a professional Chef, either in-person or virtually.

The curriculum used in this course for online learning material was licensed by Health Meets Food, a Culinary Medicine Program website utilized by more than 60 academic medical centers. This database consists of modules for teaching health science students about diet and lifestyle interventions important for medical providers. The required reading material in both the PDF and PowerPoint formats for students was originally provided by the Goldring Center for Culinary Medicine at Tulane University. Students then took a quiz on Culinarymedicine.org to assess their comprehension before participating in each class. The four modules used in the Culinary Medicine course for the participants in this study included Module 1 - Introduction to Culinary Medicine, Module 6 - Hypertension, Module 10 - Cancer, and Module 16 - Diet and Inflammation. To successfully complete and pass the course, students were required to attend all four sessions, complete quizzes after reading the required assignments and published articles, and complete the course survey for course credit.

### Data collection

Data from SurveyMonkey from the years 2018 through 2022 were included in the data analysis. The students who used a virtual format for all four sessions of the course were defined as having used the virtual method of the instruction cohort, while the students who used a strictly in-person, on-campus format were defined as having used the in-person method of the instruction cohort. The online survey included a total of 10 questions. For eight of the 10 questions, the students were asked to indicate their level of agreement with various statements on a five-point Likert scale (i.e., 1 = strongly disagree to 5 = strongly agree). The survey question content remained the same throughout the five years of survey responses collected, with one exception: the question about sharing meals was asked based on the method of instruction, either with classmates in-person or with roommates/family at home while virtual. The order of the questions in the surveys differed slightly but were aligned to match for data analysis.

Concerning the measure used, two out of the 10 survey questions were excluded from the data analysis. One question did not fit the same scoring system, as its optionset consisted of a “select all that applied” answer choice. The last question was a comment box for students to voluntarily give suggestions, offer recommendations to improve the course, or complain about the course. To determine if the constructs of interest were indeed being measured, principal component factor analysis was performed. Subsequently, two subscales were created based on those items that loaded uniquely (above 0.40) onto one of the two factors. These two subscales accounted for 60% of the variability in the total scores on the scale. Out of the eight possible questions, seven loaded uniquely into the subscales. The first survey question was excluded from the data analysis because its variance was separated between the two constructs. These constructs formed our outcomes of interest for this study. The “knowledge gained” subscale included five items (Table 1) comprising 33.12% of the total scale variance and a reliability in the acceptable range (Cronbach’s α = 0.74). The “Enjoyment” subscale consisted of two items (Table 2) comprising 25.55% of the total scale variance, and its reliability was within the acceptable range (Cronbach’s α = 0.77).

**Table 1:** Survey questions used in the knowledge gained construct

Knowledge Gained
Did you learn about food’s nutritional value, caloric content, serving sizes/portion control?
Did you find the journal articles a helpful addition to the PowerPoint presentations in discussing nutrition/evidence-based medicine?
Did you find the patient case-based exercises beneficial in helping you to assimilate your scientific knowledge with nutrition education for patients?
Do you believe that addition of this elective course is important in your medical education?
Do you feel more prepared to individualize your patient care regarding their lifestyle/diet?

The maximum points for each question were 5 points, and the total possible points were 25 points

**Table 2:** Survey questions used in the enjoyment construct

Enjoyment
Did you find the participation in the cooking portion of this class enjoyable/fun?
Did you find sharing the meals prepared together enjoyable/fun, and the discussions about each dish/recipe educational?

The maximum points for each question were 5 points, and the total points possible were 10 points

### Statistical analysis

The statistical analysis for this study was conducted using IBM SPSS Statistics version 28, a commonly used statistical analysis software. Descriptive statistics, such as the mean and standard deviation, were calculated for each quantitative variable. To compare the instruction methods for each survey item, chi-square analysis and 95% confidence intervals were used to compare participant responses between virtual and in-person instructions. The chi-square test was used because both variables were categorical (virtual vs. in-person) or ordinal (Likert scale). Independent samples one-sided t tests were used to assess differences in the subscale total scores for Knowledge Gained and Enjoyment between the instruction methods, with a significance level set at *p* <.05. This analysis was used since there was only one independent variable with two levels (virtual vs. in-person), and the dependent variables were continuous because the total scores were used for each.

Additionally, one-way ANOVA was used to analyze participant responses for Knowledge Gained and Enjoyment based on course year. ANOVA was used because there was one independent variable with five levels (cohort years) and two continuous dependent variables. Post hoc analysis was conducted using Tukey’s significant difference test, which is a commonly used post hoc method that adjusts for significance based on multiple comparisons. These statistical methods were employed to provide a comprehensive analysis of the data and determine any significant differences in outcomes based on the instruction method and course year.

## Results

A total of 360 out of 430 participants, spanning the years 2018 to 2022, successfully completed the course requirements and participated in the online survey used for data analysis. Among these participants, the valid sample size for the in-person instruction group was (N) 249, while the virtual instruction group had a valid sample size (N) 111. The knowledge gained construct consisted of five survey questions, for a total possible score of 25, while the enjoyment construct consisted of two survey questions, for a total possible score of 10. On average, the scores for each question, regardless of the instruction method, were calculated to be above 4, indicating responses between “agree” and “strongly agree.”

When comparing the data across class years, a statistically significant difference in knowledge gained was identified by one-way ANOVA (Table 3), *F* (4, 355) = 3.85, *p* =.004. Additionally, there was a statistically significant difference in enjoyment of the course between class years, *F* (4, 356) = 11.98, *p* <.001. A post hoc Tukey test* further revealed that students reported more knowledge gained in 2018, 2021, and 2022 than in 2019 (*p* =.006, 0.042, and 0.049, respectively); moreover, there was no statistically significant difference between 2020 and 2019 (*p* =.698). In terms of enjoyment, students reported more enjoyment in 2018, 2019, 2021, and 2022 than in 2020 (*p* <.001, 0.001, 0.016, < 0.001, respectively) and more enjoyment in 2021 than in 2018 (*p* =.003).

**Table 3:** ANOVA Summary for Knowledge Gained and Enjoyment in In-person and Virtual Instruction Groups (*n* = 249 and *n* = 111, respectively)

		Sum of Squares	df	Mean Square	F	*p*	partial η2	partial η2 95% CI [LL, UL]
Knowledge Gained	Between Groups	66.244	4	16.561	3.853	0.004	0.04	[0.01, 0.08]
	Within Groups	1525.712	355	4.298				
Enjoyment	Between Groups	52.256	4	13.064	11.977	0.000	0.12	[0.06, 0.18]
	Within Groups	388.303	356	1.091				

*Results of the post hoc Tukey analyses are provided in the supplementary (Table 1) material

A Chi-square test for independence was conducted to determine if there were significant differences between the students’ ratings of each item for the in-person and virtual instruction methods for the course. In Table 4, for each survey question, the top row shows data for in-person classrooms, and the bottom row shows the data for the virtual classroom. Of the seven questions asked, four had statistically significant results, while the other five did not significantly differ between the two methods of instruction. Question 1 in the survey was unique because it included aspects of both knowledge and enjoyment constructs; therefore, only a chi-square test was performed for the results of this question.

**Table 4:** Chi-Squared Analysis of Survey Responses in In-person and Virtual Instruction Groups (*n* = 249 and *n* = 111, respectively)

Survey Question	Strongly Disagree	Disagree	Neither Agree nor Disagree	Agree	Strongly Agree	Pearson Chi-Square (a = 0.05)
Did you feel that you got adequate training on new culinary techniques (slice, dice, julienne, & etc.) and kitchen safety?	0.0%	2.8%	4.4%	33.7%	59.0%	0.030*
	0.9%	4.5%	10.7%	38.4%	45.5%	
Did you learn about food’s nutritional value, caloric content, serving sizes/portion control?	0.0%	0.4%	0.4%	19.7%	79.5%	0.844
	0.0%	0.0%	0.9%	20.5%	78.6%	
Did you find the participation in the cooking portion of this class enjoyable/fun?	0.0%	0.8%	0.8%	11.6%	86.7%	0.000**
	0.9%	2.7%	6.3%	31.3%	58.9%	
Did you find sharing the meals prepared together (alongside chef either in-person or virtually) enjoyable/fun, and the discussions about each dish/recipe educational?	0	0.4%	1.6%	14.1%	83.9%	0.000**
	0	2.7%	12.5%	29.5%	55.4%	
Did you find the journal articles a helpful addition to the PowerPoint presentations in discussing nutrition/evidence-based medicine?	0	1.6%	17.3%	41.0%	40.2%	0.168
	0	2.7%	8.9%	41.1%	47.3%	
Did you find the patient case-based exercises beneficial in helping you to assimilate your scientific knowledge with nutrition education for patients?	0.8%	0.4%	4.0%	22.9%	71.9%	0.971
	0.9%	0.0%	3.6%	22.3%	73.2%	
Do you believe that addition of this elective course is important in your medical education?	0	0.8%	0.8%	21.3%	77.1%	0.088
	0	0.0%	3.6%	27.7%	68.8%	
Do you feel more prepared to individualize your patient care regarding their lifestyle/diet?	0.0%	0.9%	3.6%	42.3%	53.2%	0.022
	0.0%	0.9%	3.6%	42.3%	53.2%	

*KEY*: blue = in-person; white = virtual

*P <.05 is significant; **P <.001 is highly significant

T-tests were used to compare the mean scores for knowledge gained and enjoyment between the in-person and virtual instruction methods (Table 5). The results showed that there was no statistically significant difference in knowledge gained between the in-person and virtual instruction methods, *t* (358) = 0.53, *p* =.299. However, there was a statistically significant difference in enjoyment between the two methods (*t* (138.87) = 5.68, *p* <.001) after accounting for unequal variances, with Cohen’s d equal to 0.807, indicating a large effect size. These findings suggested that participants in the in-person instruction method group reported greater enjoyment than did those in the virtual method group.

**Table 5:** Group statistics for the dependent variable based on the instruction method

Dependent Variable	Instruction method	N	Mean	Std. Deviation
Knowledge Gained	In-Person	249	23.0281	2.07986
	Virtual	111	22.9009	2.16986
Enjoyment	In-Person	249	9.6586	0.76723
	Virtual	112	8.8214	1.47174

## Discussion

The study uncovered a consistent and high level of satisfaction among students who completed the Culinary Medicine course over fiveyears. The majority expressed satisfaction with the knowledge gained and overall enjoyment of the course, as reflected in the survey responses. Notably, the 360 students who completed the course expressed contentment with the acquired information and skills. Despite not being a graduation requirement for the Osteopathic Medical Program, students recognized its importance for future clinical practice as physicians, highlighting the course’s perceived value beyond mandatory academic obligations.

A comparison of virtual and in-person instructions for the Culinary Medicine course revealed that the in-person group enjoyed the course more significantly than did the virtual group. However, there was no significant difference in the knowledge gained between the two formats. This aligns with the finding of Brennan et al., who observed that the online and semi online (mixed-mode) cooking experiences in a course yielded comparable benefits to the traditional, in-person cooking experience conducted in a kitchen lab [10]. This study’s finding suggested that, for preclinical medical education in culinary medicine, virtual instruction might be less preferred for greater enjoyment. However, integrating virtual components, including reading assignments, quizzes, and PowerPoint presentations, could still enhance knowledge acquisition. In contrast, other studies present a positive perspective on virtual culinary teaching. Several authors [11] argue that programming in a virtual environment can enhance the learning experience. This positive effect may arise from increased self-efficacy, as participants engage in the coursework within their home kitchens. Additionally, the virtual setting allows students to utilize class time for meal preparation throughout the week. Poulton et al. emphasized that virtual technology removes limitations imposed by the absence of a teaching kitchen. Teaching students to cook in their own kitchens is seen as potent, empowering them to utilize the available space and tools effectively. This approach also encourages students to visit grocery stores, procuring ingredients they might not have considered purchasing before [9].

The decrease in perceived enjoyment in 2020 is attributed to the substantial transition toward a virtual learning environment prompted by the global pandemic. The challenges associated with this transition, such as adapting to remote instruction, potential technological issues, the absence of in-person interactions and maintaining the quality of culinary teaching in a virtual setting, as well as a potential lack of student engagement, can impact the overall learning experience. The factors contributing to the lower ratings in 2019 remain unidentified, and despite initial examination of the comments of the course survey, a more detailed understanding could not be obtained.

A limitation of this study that could have influenced the results is the difference in the level of hands-on experience between the virtual and in-person instruction formats. In the virtual classroom, students were not required to cook alongside the Chef on the virtual platform, which may have affected their responses regarding culinary techniques and enjoyment of the cooking portion of the course. The extent to which virtual students practiced their culinary skills is unknown, and this could have impacted their agreement responses on the survey. In contrast, the in-person cooking classroom allowed students to make multiple recipes alongside their classmates, with the Chef available for questions and guidance. This hands-on approach likely contributed to the in-person students’ stronger agreement with the enjoyment of the course and perceived knowledge gained about culinary skills and nutrition. In addition, virtual class students were required to purchase their own ingredients, whereas in-person, they were provided with the ingredients. This is a potential limiting factor in whether the virtual students participated in the recipe portion of the course. Specifically, during the pandemic, where the course was virtual, grocery store shopping was limited due to exposure precautions.

While self-selection bias is acknowledged as a confounding factor and limitation in the current study on culinary medicine, there is a need to explore this topic with a more diverse participant pool. Research studies that incorporate culinary medicine as a mandatory curriculum requirement in medical school programs can offer valuable insights. For instance, a study by D’Adamo et al. [12]. implemented a culinary medicine course as a compulsory component of the medical school curriculum, minimizing self-selection bias. In the study, participants were required to engage with culinary medicine concepts, irrespective of their individual interests. This approach allows researchers to examine the impact of culinary medicine education on a broader spectrum of medical students, providing insights into its effectiveness beyond the influence of self-selection. Findings from such studies can contribute to a more comprehensive understanding of the benefits and challenges associated with integrating culinary medicine into medical education.

It is important for future research to build upon this foundation and explore the same variables within different medical school settings, where the course is obligatory rather than elective. This shift in study design helps mitigate self-selection bias, enabling researchers to draw more robust conclusions about the potential impact of culinary medicine education on medical students’ engagement and enjoyment across diverse populations.

At minimum, recommendations for designing future culinary medicine courses include a hybrid combination of the in-person component dedicated to the cooking segment of the course. The hands-on experience of cooking alongside a chef and instructors providing step-by-step instructions is an invaluable aspect unique to culinary education. This practical exposure is fundamental for understanding the time and factors necessary to prepare healthy meals, which is essential for effectively conveying this message to future patients.

## Conclusion

The data gathered from the surveys conducted during the Culinary Medicine course suggest that students, in general, expressed satisfaction with the course. However, there were notable differences in enjoyment and perceived knowledge gained between virtual and in-person instruction formats, as well as among different class years. These findings provide valuable insights into the nuances of student experiences in the course and may serve as a foundation for further analysis and exploration of comments in the course survey to gain a more comprehensive understanding of the results.

Based on our study data, we did not find support for Hypothesis 1, which proposed a significant difference in knowledge gained between in-person and virtual instruction. Our analysis revealed no statistically significant difference in the levels of knowledge gained between the two modes of instruction. However, Hypothesis 2 was supported by our findings, as in-person students reported higher levels of course enjoyment than virtual students did, indicating that they enjoyed the course more. In terms of Hypothesis 3, our results were partially consistent with the hypothesis. In 2020, students who were in a virtual class reported lower levels of course enjoyment and knowledge than did those in other years. However, in contrast to our initial hypothesis, students in 2021, including some who were also in virtual classes, reported higher levels of satisfaction in terms of knowledge gained and course enjoyment, which contradicts our third hypothesis.

Overall, these study data suggest that students gained knowledge regardless of the method of teaching (in-person) but that students who tended to enjoy the course more often were taught in-person. Additionally, the impact of virtual instruction on course enjoyment and knowledge gained may vary across different years, as evidenced by the mixed findings in 2020 and 2021. However, further research may be needed to explore the underlying factors contributing to these results.

In conclusion, Culinary Medicine course survey data provide valuable insights into students’ experiences and perceptions of the course. While students generally expressed satisfaction with the course, there were differences in enjoyment and perceived knowledge gained between virtual and in-person instruction formats, as well as among different class years. The potential limitations related to required reading and hands-on experience in the virtual classroom should be taken into consideration when interpreting the findings. This study may be useful for further research when comparing virtual and in-person methods of instruction in medical school curricula, such as laboratory courses that involve hands-on portions. Further analysis and exploration of comments in the course survey can provide additional insights into the reasons behind these differences and inform potential improvements for future iterations of the course. Overall, the findings contribute to the growing field of Culinary Medicine and highlight the importance of considering various factors that can impact student experiences in a medical education program that integrates culinary skills and nutritional knowledge.

### Electronic supplementary material

Below is the link to the electronic supplementary material.

Supplementary Material 1

## Data Availability

The datasets used and/or analyzed during the current study are available from the corresponding author upon reasonable request.

## Abbreviations

PCOM: Philadelphia College of Osteopathic Medicine
D.O.: Doctor of Osteopathic Medicine
N: Valid sample size
