# Supplementary material for: Exploring the effectiveness of virtual and in-person instruction in culinary medicine: a survey-based study
Source: BMC Med Educ. 2024 Mar 13;24:276. doi: 10.1186/s12909-024-05265-w (PMC10935775; doi:10.1186/s12909-024-05265-w)
Supplement: Supplementary file 1 — Supplementary Material 1 [file 12909_2024_5265_MOESM1_ESM.pdf]

Supplemental Table 1: Tukey Post-Hoc Test Analysis

Dependent Variable	Year	Years compared	Mean Difference	Standard Deviation	Sig. a =0.05
Knowledge Gained	2019	2018	-1.413	0.413	0.006**
		2020	-0.536	0.416	0.698
		2021	-1.110	0.395	0.042*
		2022	-1.094	0.398	0.049*
Enjoyment	2018	2019	0.295	0.208	0.617
		2020	1.120	0.176	0.000
		2021	0.601	0.164	0.003**
		2022	0.241	0.166	0.595
	2020	2018	-1.120	0.176	0.000**
		2019	-0.825	0.209	0.001**
		2021	-0.519	0.166	0.016**
		2022	-0.878	0.167	0.000**

*P<.05 is significant; **P< .001 is highly significant
